# Creative Health Associates and the Two Loops model of systems change: key insights for the field

**DOI:** 10.1177/17579139261436767

**Published:** 2026-05-27

**Authors:** J Hearst, J Howard, G Buckland, B Crosland

**Affiliations:** Research and Policy Manager, National Centre for Creative Health, Oxford, UK; Honorary Research Fellow, University College London, London, UK; Programme Manager, National Centre for Creative Health, PO Box 948, Oxford OX1 9TY, UK; Co-director of Justice Futures CIC, Do It Justice, Kent, UK; Evaluation and Learning Specialist, Freelance, London, UK

## Abstract

In this article, the authors show how important it is to have people in bridging roles who can connect different sectors and explain the value of creativity in terms that matter to health leaders. By supporting pioneers, sharing evidence, and building new partnerships, Creative Health Associates have laid the foundations for creative health to become part of mainstream public health approaches.

From July 2023 to March 2025, the National Centre for Creative Health (NCCH) hosted an Arts Council England–funded *Creative Health Associates* (CHA) programme,^1^ placing a CHA in every NHS region in England. Acting as bridges between sectors, CHAs embedded into integrated care systems (ICSs) to advance understanding of creative health and support systems change.

An external evaluation used the Two Loops model of systems change^2^ to show how the programme contributed to the emergence of new systems. This article summarises the evaluation’s key public health insights.

## Overview

The CHA programme demonstrated that change in public health systems is rarely linear. Instead, it unfolds in iterative, interconnected cycles across multiple levels, which is exactly the type of complex environment that the Two Loops model was designed to help navigate.^3^

Analysis of the programme showed that much of the impact achieved by CHAs in public health settings sits within the early and bridging phases of the Two Loops – the space where groundwork is laid, language is shaped, and relationships are built to support deeper systemic transformation. However, some impact was achieved throughout the whole Two Loops life cycle.

## Incumbent System – Making The Invisible Visible

Before CHA involvement, public health teams often had little direct contact with cultural teams, despite their shared objectives on tackling inequalities – both cultural access and health outcome. In several regions, CHAs uncovered extensive creative health activity that had gone unnoticed by public health professionals.

**Figure:**
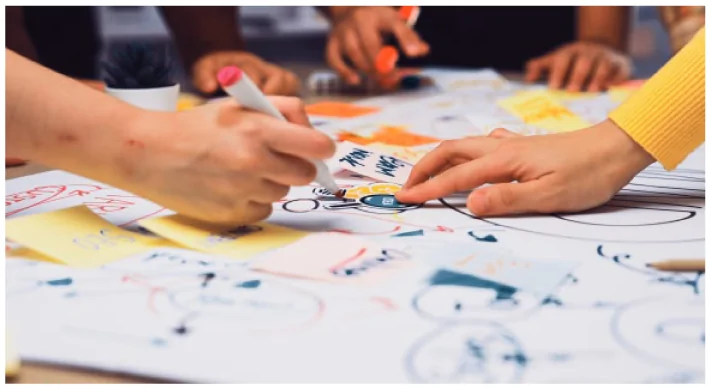


By naming and presenting these activities and stakeholders as public health assets, CHAs reframed them as enablers for prevention and wellbeing. The very existence of the CHA post, particularly when supported by public health teams, lent formal credibility to the agenda and opened new dialogue with health inequalities leads.

In Two Loops terms, this work represents the transition from incumbent system to the dual early phases of *naming* and *connecting*, whereby CHAs made early innovators visible and linked them to decision-makers.

## Naming – Language As A Lever

In most areas, the term ‘creative health’ was unfamiliar to public health audiences. CHAs introduced and normalised the term as a shared strategic language, shifting the conversation from fragmented activity to a coherent prevention-focused framework.

Naming also involved tailoring the evidence on creative health to resonate with local public health priorities. This framing allowed public health leaders and population health managers to articulate and defend creative health as part of their strategies.

Importantly, in the Two Loops model, *naming* shapes the emerging system’s narrative so that early adopters can recognise their work within it and identify its strategic potential.

## Connecting – Building Cross-Sector Alliances

CHAs convened a number of place-based, cross-sector groups that brought together public health professionals, cultural organisations, clinical leaders, and community partners. Acting as *systems convenors*^4^ in spaces where health inequalities manifest, they uncovered duplication of effort and redirected energy towards more strategic use of resources. These alliances created shared ownership of creative health initiatives and positioned them within the wider determinants of health agenda.

In Two Loops terms, *connecting* moves isolated actors into a community of practice that can gain momentum and resist being pulled back into established patterns.

## Pockets Of The Future – Strategic Embedding

As relationships deepened, creative health began to appear in public health agendas, learning events, and health inequalities strategies developed through Integrated Care Board (ICB)–public health collaborations. Public health leaders increasingly viewed creative health as a transdisciplinary prevention tool rather than a supplementary activity. In some regions, CHAs laid the foundations for peer-learning and co-design networks where public health, culture, and clinical services could innovate together alongside lived experience experts.

These pockets of practice could be considered early manifestations of a new paradigm, embedded within existing structures and demonstrating viability to a wider audience.

## Bridging – Linking Old And New

One of the CHAs’ most valuable contributions was acting as translators between the highly governed frameworks of health systems and the more fluid practices of community and arts sectors. They bridged gaps by repurposing existing roles and relationships – for example, collaborating with universities so that future public health professionals encounter creative health during their training.^5^ Bridging also meant framing creative health in terms of system priorities, such as addressing cost drivers. As the NHS considers more public health approaches and priorities, as a consequence of the 10-year plan,^6^ these bridging roles are arguably more relevant than ever.

In the Two Loops model, the purpose of *bridging* is to preserve useful aspects of the old system while feeding resources and credibility into the new.

## Nourishing – Sustaining Early Adopters

Public health champions of creative health are often isolated within their systems. CHAs provided a crucial source of support, amplifying their voices, connecting them with like-minded peers, and supplying them with evidence and practical resources to strengthen their case.

**Figure 1:**
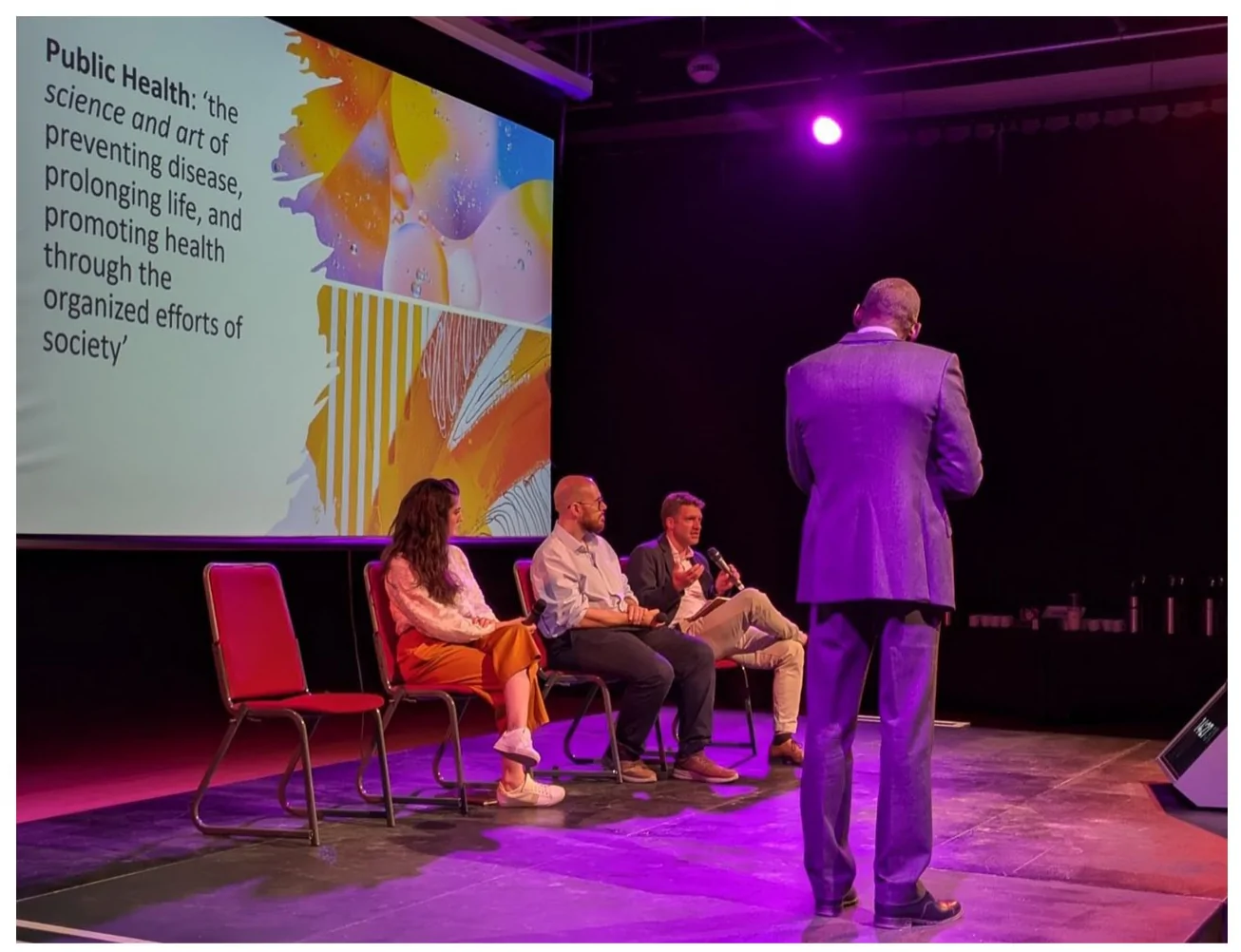
Panel 1 – *Perspectives from Directors of Public Health*^9^ – from *The Place of Creativity in Public Health* event, September 2024; one of four events to feature in the Midlands Roundtable series.

In some cases, CHAs platformed Directors of Public Health and other public health professionals who were already engaged with creative health, enabling them to influence regional or national discussions (Figure 1). Resource packs, evidence summaries, and tailored briefing materials acted as ongoing nourishment to keep momentum alive in commissioning and policy spaces.^7,8^

Within the Two Loops framework, *nourishing* sustains pioneers until the emerging system becomes resilient enough to self-sustain.

## Illuminating – Shaping The Public Health Narrative

At the more advanced end of the change spectrum, CHAs helped position public health–led creative health initiatives on the national stage, reframing them as replicable models. They developed targeted evidence narratives^10^ for specific public health needs, strengthening both emotional and strategic arguments for adoption. This visibility began to shift perceptions of creative health from a ‘nice-to-have’ to a strategically relevant component of prevention and health inequalities work.

In Two Loops terms, this is the *illuminating* phase – making the new paradigm the visible and credible choice.

## Key Takeaways

The CHA programme’s impact on public health illustrates the power of the Two Loops model as a planning tool for systems change. Concerns central to public health – prevention, health equity, and community-based care – are beginning to shape even traditional NHS spaces through strategies like the 10-year plan, demonstrating the relevance of this model to a range of creative health developments.

For leaders seeking to embed creative health as a mainstream prevention strategy, the Two Loops model offers a way to identify where energy is already building, where bridges are needed, and how to sustain early adopters until systemic change becomes the norm. The CHA programme demonstrates the importance of bridging roles in catalysing this change.
